# Incorporation of CEUS and SWE parameters into a multivariate logistic regression model for the differential diagnosis of benign and malignant TI-RADS 4 thyroid nodules

**DOI:** 10.1007/s12020-023-03524-2

**Published:** 2023-10-27

**Authors:** Hong-Jing Li, Guo-Qing Sui, Deng-Ke Teng, Yuan-Qiang Lin, Hui Wang

**Affiliations:** Department of Ultrasound, China-Japan Union Hospital of Ji Lin University, Changchun, Jilin, China

**Keywords:** Ultrasonography, Thyroid gland, ACR TI-RADS, Time–intensity curve (TIC)

## Abstract

**Purpose:**

To investigate the diagnostic value of contrast-enhanced ultrasound (CEUS) quantitative analysis parameters combined with shear wave elastography (SWE) quantitative parameters in the differentiation of benign and malignant ACR TI-RADS category 4 thyroid nodules and to provide a more effective reference for clinical work.

**Methods:**

We analyzed 187 category 4 nodules, including 132 nodules in the development cohort and 55 nodules in the validation cohort, divided the development cohort into benign and malignant groups, and analyzed the differences in all CEUS and SWE quantitative parameters between the two groups. We selected the highest AUC of the two parameters, performed binary logistic regression analysis with the ACR TI-RADS score and constructed a diagnostic model. ROC curves were applied to evaluate their diagnostic efficacy.

**Results:**

1) The diagnostic model had an AUC of 0.926, sensitivity of 87.5%, specificity of 86.8%, diagnostic threshold of 3, accuracy of 87.12%, positive predictive value of 86.15%, and negative predictive value of 88.06%. 2) The diagnostic model had an AUC of 0.890 in the validation cohort, sensitivity of 81.5%, specificity of 79.6%, and accuracy of 80.00%.

**Conclusion:**

The combined multiparameter construction of the nodule diagnostic model can effectively improve the diagnostic efficacy of 4 types of thyroid nodules and provide a new reference index for clinical diagnostic work.

## Introduction

Along with the gradual increase in public health awareness, thyroid nodules are becoming more widespread in the public. Thyroid nodules can be detected in 19–68% of the population, and the detection rate of nodules is also significantly higher [1]. Characterized by noninvasiveness, repeatability, low cost, and convenience, high-frequency ultrasound has become the preferred screening method for thyroid nodules, showing its great reference value in clinical decision-making and its value as a guide in the qualitative diagnosis and treatment of thyroid nodules. To standardize the diagnostic criteria for thyroid nodules and reduce diagnostic heterogeneity, the American College of Radiology (ACR) published the White Paper on Thyroid Imaging Reporting and Data System (TI-RADS) [2] (hereinafter referred to as ACR TI-RADS) in 2017. In ACR TI-RADS, thyroid nodules are classified into classes 1–5 based on a weighted score made up of different characteristics. The diagnostic features of ACR TI-RADS category 3 and 5 nodules are distinct, and the nature of the nodule is easily diagnosed by routine ultrasound. However, the ultrasound features of category 4 nodules overlap to some extent, making the diagnosis of benign or malignant nodules more difficult [3–6]. Fine-needle aspiration (FNA) is one method of diagnosing the nature of thyroid nodules and is an invasive diagnostic method [7]. Sonographers and clinicians are constantly exploring noninvasive means to improve the diagnostic efficacy for TI-RADS 4 nodules. With the ongoing development of different examination techniques, a variety of new ultrasound techniques have been incorporated into the diagnosis of thyroid nodules. Contrast-enhanced ultrasound (CEUS) has been widely used in clinical work due to its unique blood pool technique. Its contrast agent has few side effects and can be excreted with the pulmonary circulation. Contrast-enhanced ultrasound shows great clinical reference value in the diagnosis of breast, liver, and kidney diseases [8–10] and high diagnostic efficacy for the nature of thyroid nodules [11, 12], so it has played an increasingly important role in the diagnosis of thyroid nodules [13]. However, the results of thyroid nodule contrast perfusion are still determined by ultrasound sonographers, making the results subjective. Vue Box^@^, an external perfusion analysis software, can visually display the quantitative parameters of contrast perfusion in lesions [14], and then quantitative analysis can be conducted, which is more objective and can effectively avoid the subjectivity of sonographers in the diagnosis of thyroid nodules.

Shear-wave elastography (SWE) is a new quantitative diagnostic method that has advantages over strain rate elastography (a semiquantitative diagnostic method). It can more intuitively display the elastic characteristics of thyroid tissues inside and around nodules, making for better diagnostic performance for thyroid nodules [15, 16]. Some shortcomings of SWE are that its measured values will be affected by calcification in the nodule, and there are high skill requirements for the operator. Therefore, a more objective diagnostic method for diagnosing thyroid nodules is needed. Few studies have been reported on the application of quantitative ultrasonography parameters combined with quantitative shear wave elastography parameters to construct a predictive model to improve the detection rate of malignancy in f category 4 nodules. This study constructed a diagnostic model for TI-RADS 4 nodules using the quantitative parameters of CEUS and SWE, aiming to improve the diagnostic efficacy for TI-RADS 4 nodules by a noninvasive method and develop a more valuable reference for clinical work.

## Materials and methods

### Study oversight

This study was approved by the ethics committee. All patients signed an informed consent form prior to surgery and were informed of the risks associated with the procedure.

### Patients

A total of 187 patients (187 nodules) who were treated in the Department of Ultrasound of our hospital from January 2021 to October 2021 and underwent ultrasonography, CEUS and SWE with definite pathological results were enrolled, including 132 patients (132 nodules) in the development cohort and 55 patients (55 nodules) in the validation cohort. There were 49 males and 138 females aged 19–79 (46.63 ± 10.69) years, and the maximum diameter of a nodule was 4.1–31.9 (7.40 ± 3.69) mm.

The inclusion criteria were set as follows: 1) ACR TI-RADS 4 nodules (4–6 points) with definite FNA results or surgical pathological results, 2) age ≥18 years, and 3) complete ultrasonography data. The exclusion criteria were set as follows: 1) intolerance to contrast agent, 2) history of thyroid surgery or related radiotherapy, and 3) intolerance of puncture or operation.

### Instruments, methods and data analysis

An ultrasonic diagnostic instrument (Aixplorer V, Supersonic Imagine, France) with a linear-array probe (2–10 MHz and 4–15 MHz) was used, and ultrasound examination and image acquisition were carried out by two thyroid ultrasound examiners with more than 10 years of experience.

**Fig. 1:**
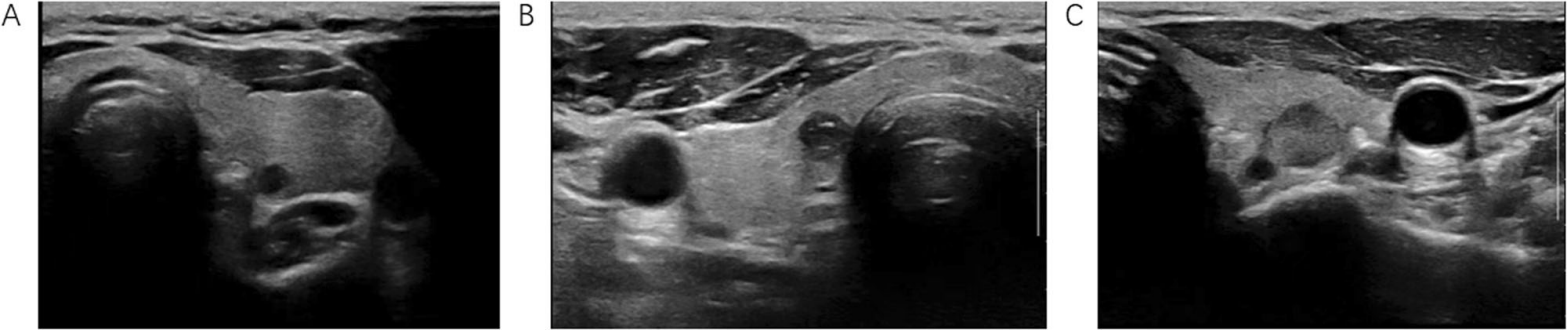
ACR4 points: low echo in the left lobe, clear boundaries, and a regular shape (**A**). ACR5 points: low echo in the right lobe, clear boundaries, a regular shape, and calcification inside (**B**). ACR6 points: low echo in the left lobe, clear boundaries, and an irregular shape (**C**)

Two-dimensional ultrasound image acquisition was conducted as follows. The patient was placed in a supine position with neck hyperextension to fully expose the thyroid region and was continuously scanned at multiple sections. The nodule position, nodule size (maximum diameter), nodule composition, echo, aspect ratio, margin, and strong echo were recorded. The nodules were given scores in accordance with the ACR TI-RADS scoring criteria [2]: composition (cystic and spongy: 0 points; mixed cystic-solid: 1 point; solid and almost completely solid: 2 points), echo (no echo: 0 points; hyperecho/isoecho: 1 point; hypoecho: 2 points; very hypoecho: 3 points), aspect ratio (<1: 0 points; ≥1:3 points), margin (smooth and unclear: 0 points; irregular lobulation: 2 points; extra thyroid invasion: 3 points), and strong echo (no/large comet tail sign: 0 points; coarse calcification: 1 point; marginal or annular calcification: 2 points; punctate strong echo: 3 points). According to the above scoring criteria, nodules with 4–6 points were defined as ACR TI-RADS 4 nodules, as shown in Fig. 1 (A: ACR 4 points; B: ACR 5 points; C: ACR 6 points).

**Fig. 2:**
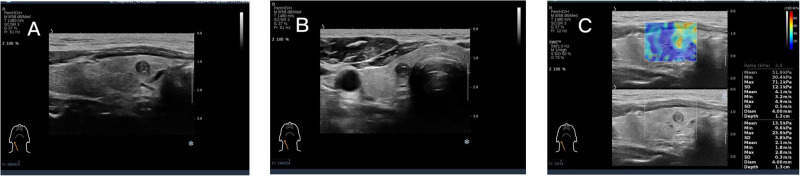
Right lobe hypoechoic 2D image (**A**). Hypoechoic SWE image of the right lobe (**B**). Hypoechoic SWE measurements of the right lobe, Emax=71.1 kPa (**C**)

SWE was performed at the maximum longitudinal section of nodules selected under two-dimensional conditions. In brief, the probe was placed gently on the neck, without compression, and the patient was instructed to hold his/her breath. With 2-mm sampling frames, the area of the hardest nodules and the area of normal thyroid tissues at the same level were compared. Then, the maximum Young’s modulus (Emax), mean Young’s modulus (Emean), minimum Young’s modulus (Emin), and elasticity standard deviation (Esd) of the nodules were detected three times, and the average values were taken. The preserved images are shown in Fig. 2.

**Fig. 3:**
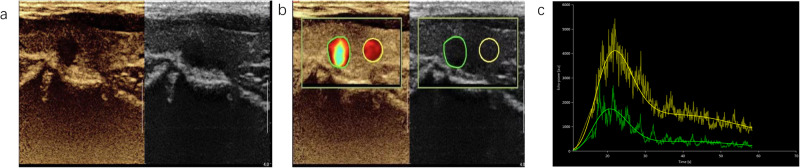
Contrast-enhanced ultrasound image of nodules (**a**). Quantitative analysis image of the left lobe nodule (**b**). TIC (yellow: normal thyroid tissues, green: nodules) (**c**)

CEUS was performed as follows: After acquisition of the shear-wave elastography image, the instrument was switched to contrast mode, and Sono Vue contrast agent was used. The freeze–dried powder vial was injected with 5 mL of 0.9% sterile sodium chloride injection and shaken until the freeze–dried powder was completely dispersed and dissolved. The SonoVue microbubble suspension was aspirated into a syringe and immediately injected into the peripheral vein (2.4 mL per injection). Then, 5 mL of 0.9% sterile sodium chloride was injected. The status of contrast agent perfusion in thyroid nodules was observed and video-recorded for 2 min. The image was then exported in DICOM format and analyzed offline in Vue _Box@._ From the time–intensity curve (TIC), the following parameters were obtained: peak enhancement (PE), wash-in rate (WiR), wash-out rate (WoR), wash-in area under the curve (WiAUC), wash-out area under the curve (WoAUC), wash-in and wash-out area under the curve (WiWoAUC), and wash-in perfusion index (WiPI). Moreover, the differences in these parameters between normal thyroid tissues and nodules were calculated: ΔPE, ΔWiR, ΔWoR, ΔWiAUC, ΔWiWoAUC, ΔWoAUC, and ΔWiPI (the analysis procedure is shown in Fig. 3).

### Statistical methods

IBM SPSS Statistics 26.0 was used for statistical analysis. Measurement data of normally distributed variables are described as $\overline x$ ± *s*, and those of nonnormally distributed variables are described as *M* (*Q1*, *Q3*). Qualitative data are expressed as *n* (%). The diagnostic efficacy was evaluated by receiver operating characteristic (ROC) curves, and diagnostic cutoff values were screened using Youden’s index. The contrast-enhanced ultrasound parameter with the largest AUC was selected, as was SWE, and these parameters were subjected to binary logistic regression analysis along with the ACR TI-RADS 4 score. From these, the diagnostic model for benign and malignant TI-RADS 4 thyroid nodules was constructed, and its AUC was compared with the individual AUCs by the *Z* test. *p* < 0.05 was considered statistically significant.

## Results

### Construction of the logistic diagnostic model

#### General basic information of patients in the development cohort

There were 132 nodules, including 68 benign nodules and 64 malignant nodules, in the development cohort (Table 1).

**Table 1:** General information of patients in the development cohort

Factor	Benign (*n* = 68)	Malignant (*n* = 64)	Statistic
TI-RADS			29.917
4 points	47 (35.6%)	14 (10.6%)	–
5 points	13 (9.8%)	27 (20.5%)	–
6 points	8 (6.1%)	23 (17.4%)	–
Emax	31.90 (26.82,35.85)	48.30 (35.02,64.58)	−6.25
Emean	22.59 (17.83,27.60)	34.90 (22.53,46.45)	−5.20
Emin	13.60 (8.93,23.25)	18.05 (10.48,28.20)	2.03
Esd	3.90 (2.20,5.90)	6.20 (3.60,9.00)	−3.79
PE	37.96 ± 3.94	34.45 ± 3.55	−5.35
WiAUC	45.53 ± 4.13	41.96 ± 3.76	−5.17
WiR	31.73 (28.89,34.42)	28.48 (25.44,31.94)	−3.21
WiPI	35.82 ± 4.13	32.46 ± 3.62	−4.94
WoAUC	45.54 ± 4.10	41.96 ± 3.76	−5.20
WiWoAUC	46.98 (44.72,49.66)	43.99 (41.53,46.65)	−4.70
WoR	28.97 (26.91,31.11)	25.86 (23.10,29.11)	−3.98
ΔPE	−0.62 (−1.53, 0.03)	3.01 (1.79,4.89)	−6.31
ΔWiAUC	−0.29 (−1.34,1.40)	3.15 (1.57,4.88)	−5.78
ΔWiR	−1.10 (−2.08,0.78)	2.93 (0.97,5.27)	−6.18
ΔWiPI	−0.65 (−1.53,0.16)	3.01 (1.61,4.93)	−6.16
ΔWoAUC	−0.29 (−1.23,1.41)	3.15 (1.58,4.88)	−5.85
ΔWiWoAUC	−0.27 (−1.31,1.24)	3.23 (1.59,4.99)	−6.14
ΔWoR	−0.93 (−2.32,1.06)	2.91 (1.11,5.21)	−5.97

#### Shear-wave elastography parameter analysis

**Fig. 4:**
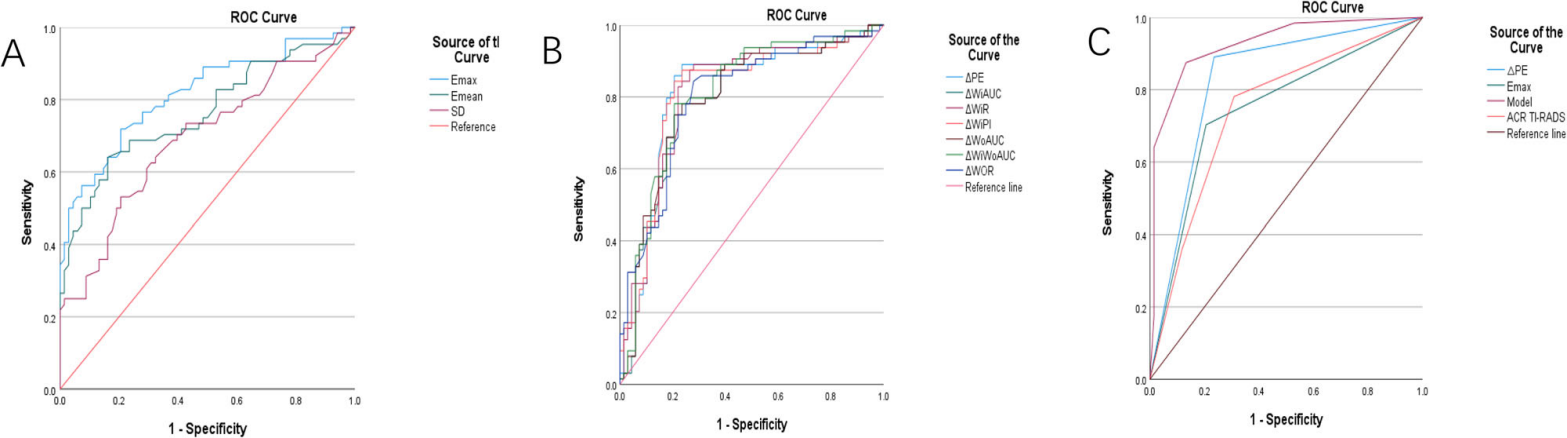
ROC curves of SWE parameters in the diagnosis of benign vs. malignant thyroid nodules (**A**). ROC curves of CEUS quantitative parameters in the diagnosis of benign vs. malignant thyroid nodules (**B**) (Δ: difference values of parameters between normal thyroid tissues and nodules). ROC curves of ΔPE, Emax, ACR-TIRADS, and the logistic model (development cohort) (**C**)

Unlike Emin (*p* ≥ 0.05), Emax, Emean, and Esd were significantly different between the benign and malignant groups (*p* < 0.05). As shown by the ROC curves, the AUCs of Emax, Emean, and Esd were 0.816, 0.763, and 0.687, respectively. Emax had the largest AUC, with a cutoff of 36.00 kPa, sensitivity of 72.90%, specificity of 79.40%, positive predictive value of 75.40%, and negative predictive value of 74.6% (Fig. 4A).

#### CEUS parameter analysis

The contrast images were analyzed using Vue Box^@^, and the TIC was automatically plotted by the instrument. Statistical analyses were conducted on the parameters PE, WiR, WoR, WiAUC, WiWoAUC, WoAUC, and WiPI of the nodules, as were their difference values ΔPE, ΔWiR, ΔWoR, ΔWiAUC, ΔWiWoAUC, ΔWoAUC, and ΔWiPI between nodules and normal thyroid tissues. They all displayed statistically significant differences between the two groups (*p* < 0.05). As shown by the ROC curves, ΔPE had the highest diagnostic efficacy (AUC = 0.819), with a cutoff of 0.16 dB, sensitivity of 89.10%, and specificity of 76.50% (Fig. 4B).

#### Results of binary logistic regression analysis

With the pathological result as the dependent variable and Emax, ΔPE, and the ACR TI-RADS score as independent variables, binary logistic regression analysis was carried out. The results showed statistically significant differences between the benign and malignant groups (*p* < 0.05) (Table 2).

**Table 2:** Results of multivariate logistic regression analysis

Factor	*β*	SE	*χ*^*2*^	*df*	*p*	OR	95% CI	
							Lower limit Upper limit	
TI-RADS			17.427	2	<0.001			
4 points						1		
5 points	1.606	0.632	6.448	1	0.011	4.982	1.442	17.206
6 points	2.688	0.759	12.536	1	<0.001	14.707	3.321	65.139
Emax								
<36 kPa	1.732	0.551	9.867	1	0.002	1	1.918	16.647
≥36 kPa						5.650		
∆PE								
<0.16db	3.214	0.622	27.546	1	<0.001	1	7.492	82.635
≥0.16db						24.882		
Constant	−3.975	0.729	29.750	1	<0.001	0.019		

#### Construction of the logistic regression diagnostic model and evaluation of its diagnostic efficacy

According to the β value in the binary logistic regression analysis, the standardized regression coefficients of the above parameters were calculated and rounded 2-fold. Then, the scores were added to construct the logistic diagnostic model, and the ROC curve was plotted to evaluate the diagnostic efficacy of the model. The AUC, diagnostic cutoff value, sensitivity, and specificity of the diagnostic model were 0.926, 3 points, 87.5%, and 86.8%, respectively. When the diagnostic cutoff value was ≥3 points, the accuracy, positive predictive value, and negative predictive value of the diagnostic model were 87.12, 86.15, and 88.06%, respectively. The scoring criteria are shown in Table 3, and the ROC curve of the diagnostic model is shown in Fig. 4C.

**Table 3:** Logistic scoring method assignment table

Factor	Assignment
ACR TI-RADS	
4 points	0
5 points	1
6 points	2
Emax	
<36 kPa	0
≥36 kPa	1
∆PE	
<0.16db	0
≥0.16db	2

### Validation of the logistic diagnostic model

#### General information of patients in the validation cohort

There were 55 nodules, including 28 benign nodules and 27 malignant nodules, in the validation cohort (Table 4).

**Table 4:** General information of patients in the validation cohort

Factor	Benign (*n* = 28)	Malignant (*n* = 27)	Statistic
TI-RADS			13.97
4 points	20 (36.4%)	6 (10.9%)	–
5 points	6 (10.9%)	12 (21.8%)	–
6 points	2 (3.6%)	9 (16.4%)	–
Emax	32.90(26.00,40.10)	48.70(35.60,60.20)	−3.30
Emean	26.35(18.70,32.85)	34.60(23.10,41.90)	−2.57
Emin	18.26 ± 8.41	24.14 ± 13.83	1.91
Esd	4.90 (3.20,9.70)	3.75 (2.13,5.35)	−2.05
PE	38.53 ± 3.48	34.77 ± 2.69	−4.46
WiAUC	43.17 (41.61,45.48)	39.80 (37.98,41.81)	−3.67
WiR	32.30 (29.25,37.40)	29.67 (26.84,34.06)	−1.51
WiPI	36.66 ± 6.41	31.85 ± 3.53	−4.05
WoAUC	46.29 ± 3.89	42.14 ± 2.58	−4.63
WiWoAUC	47.82 (45.92,49.54)	45.02 (41.74,46.41)	−4.04
WoR	29.21 ± 4.60	27.03 ± 4.54	−1.77
ΔPE	−0.48 (−1.38,1.36)	2.57 (0.81,4.79)	−3.35
ΔWiAUC	−0.15 (−1.00,2.57)	3.15 (1.25,4.76)	−3.17
ΔWiR	−0.98 (−2.08,1.16)	3.04 (0.81,4.91)	−3.33
ΔWiPI	−0.65 (−1.51,1.15)	3.40 (0.81,5.20)	−3.58
ΔWoAUC	−0.20 (−1.21,1.61)	2.54 (0.61,5.48)	−3.50
ΔWiWoAUC	−1.30 (−1.23,1.36)	2.74 (0.37,5.47)	−3.57
ΔWoR	−0.95 (−1.68,0.78)	3.07 (0.65,5.18)	−3.59

#### Validation and comparison of the logistic diagnostic model

**Fig. 5:**
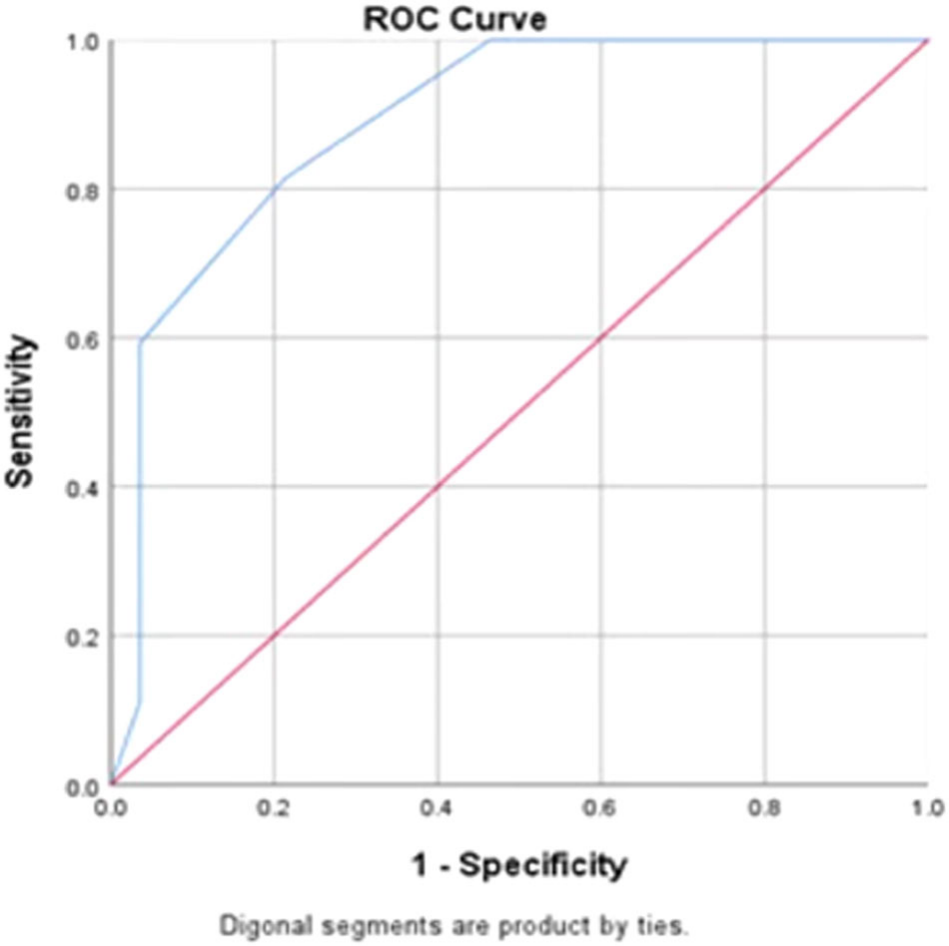
ROC curves of ΔPE, Emax, ACR-TIRADS, and the logistic model (validation cohort) ROC curves of the logistic medle (validation cohort)

The data of patients in the validation cohort were substituted into the scoring model to evaluate the diagnostic efficacy of the diagnostic model, with postoperative pathology as the gold standard. In the validation cohort, 27 nodules (49.1%) were pathologically malignant and 28 nodules (50.9%) were pathologically benign, while 22 nodules were diagnosed as malignant and 22 nodules were diagnosed as benign by the diagnostic model. The AUC, accuracy, rate of missed diagnosis, sensitivity, specificity, positive predictive value, and negative predictive value of the diagnostic model were 0.890, 80.00, 20, 81.5, 79.6, 78.57, and 81.48%, respectively, in the validation group. The model had significantly higher diagnostic efficacy, sensitivity, and accuracy than Emax, ΔPE, or ACR TI-RADS alone in diagnosing the nature of thyroid nodules. The ROC curves of the above four methods were compared by the *Z* test (Table 5, Fig. 5).

**Table 5:** Comparison of AUCs of ΔPE, Emax, ACR-TIRADS and the logistic model

Parameter	AUC (95% CI)	*p*	Sensitivity (%)	Specificity (%)	Accuracy (%)	Z
∆PE	0.783 (0.656–0.910)	<0.001	85.20	71.40	78.18	0.197
Emax	0.673 (0.529–0.818)	0.027	70.40	64.30	67.30	−3.852
TI-RADS	0.766 (0.637–0.895)	0.001	77.80	71.40	58.18	−2.155
logistic model	0.890 (0.802–0.978)	<0.001	81.50	79.60	80.00	–

## Discussion

With the continual development of high-frequency ultrasound techniques, the detection rate of nodules has been increasing yearly. To standardize the diagnostic criteria for thyroid nodules, guidelines for risk stratification of thyroid nodules have been developed by scholars from different countries. Among them, ACR TI-RADS has a lower unnecessary FNA rate than other guidelines [17, 18]. According to the ACR TI-RADS risk stratification guidelines, the probability of malignancy of thyroid nodules of category 4 is 5–20% [2], its diagnosis is still clinically difficult, and the diagnosis of this type of nodule is clinically important. Therefore, it is of more clinical significance to improve the diagnostic efficacy for ACR TI-RADS 4 nodules. CEUS and SWE can enhance the diagnostic efficacy for thyroid nodules, and the combination of the two has even better diagnostic efficacy [19]. In this study, we intend to try to apply the quantitative analysis parameters of ultrasonography and the quantitative analysis parameters of shear wave elastography to construct a predictive model for category 4 nodules, in order to ware to improve the malignancy detection rate of suspicious nodules among the category 4 nodules. In this study, a diagnostic model for ACR TI-RADS 4 nodules was constructed using contrast-enhanced ultrasound and SWE quantitative parameters and the ACR TI-RADS score. From the ROC curves, we found that the diagnostic cutoff value of the new model was 3 points. The AUC, accuracy, sensitivity, specificity, positive predictive value, and negative predictive value of the diagnostic model were 0.890, 80.00, 81.50, 79.60, 78.57, and 81.4%, respectively, in the validation cohort. The diagnostic efficacy, sensitivity, and accuracy of the diagnostic model were superior to those of any other diagnostic method. When the ACR TI-RADS score was 4 points, the diagnostic cutoff value could be reached under the condition of Emax ≥36 kPa and ΔPE ≥ 0.16 db. When the ACR TI-RADS score was 5 points, the diagnostic cutoff value could be reached under the condition of ΔPE ≥ 0.16 dB. When the ACR TI-RADS score was 6 points, the diagnostic cutoff value could be reached under the condition of Emax ≥36 kPa. The diagnostic model could effectively avoid missed diagnoses when the ACR TI-RADS score was 4 points and avoid misdiagnoses when the ACR TI-RADS score was 6 points.

Vue Box^@^ is an external offline perfusion analysis software. Its integrated correction can evaluate the wash-in and wash-out kinetics of microvessels in detail [20]. We calculated the TIC parameters through perfusion analysis. In this study, the contrast parameters selected had statistically significant differences between the benign and malignant groups (*p* < 0.05). As shown in the ROC curves, ΔPE had the highest diagnostic efficacy (AUC = 0.819), with a cutoff of 0.16 dB, sensitivity of 89.10%, specificity of 76.50%, and accuracy of 80%. Huang Y et al. [21] found that PE had good diagnostic efficacy for thyroid micronodules (≤10 mm), consistent with this study. Deng J et al. [21] also found that inhomogeneous low enhancement is a risk indicator for malignant thyroid tumors. Huang Y et al. [22] The AUC of enhancement was 0.863 (95% CI 0.807 ~ 0.907, *P* < 0.001) when applying the modified version of the TIRADS for ultrasound, which was higher than that of ACR alone (0.738 (95% CI 0.672 ~ 0.797, *P* < 0.001)) and CEUS (0.835 (95% CI 0.777 ~ 0.884, *P* < 0.001). Yan Zhang et al. [3] argued that the diagnostic efficacy, sensitivity, and specificity of CEUS combined with TI-RADS for thyroid nodules were better than those of either input alone. In this study, the diagnostic efficacy (0.926), specificity (86.80%), and accuracy (87.12%) of the multiparameter diagnostic model in the development cohort were higher than those of any single method, but its sensitivity (87.50%) was lower than that of ΔPE (89.10%), consistent with the view in EFSUMB guidelines that contrast-enhanced ultrasound is the most effective when combined with other examination methods [23].

SWE is a noninvasive method for evaluating tissue hardness. Specifically, transverse shear waves are generated by shear-wave elastography in tissues, and shear wave velocities are measured and converted into Young’s modulus. Young’s modulus can be used to directly evaluate the hardness of nodules and visually display the hardness of thyroid nodules and surrounding thyroid tissues. Boasting real-time performance, high efficiency, and repeatability, SWE has been widely applied to the clinical diagnosis of various diseases [24]. After malignant transformation, the histological characteristics of thyroid nodules are changed, such as poor elasticity and increased hardness. The hardness of nodules has been positively correlated with the degree of malignancy, and the higher the maximum Young’s modulus of a nodule is, the higher the malignancy risk [25]. The diagnostic cutoff values for thyroid nodules vary between studies [26, 27], so no standard diagnostic cutoff value of Young’s modulus for thyroid nodules has been defined in guidelines. In this study, Emax had the highest diagnostic efficacy [AUC = 0.816, vs. Emean (AUC = 0.763) and Esd (AUC = 0.687)], with a diagnostic cutoff value of 36.00 kPa, sensitivity of 72.90%, specificity of 79.40%, and accuracy of 75.00%, similar to the results of Zhao [28]. The cutoff value of Emax in this study was far higher than that (18.2 kPa) in the study of Petersen [29] and far lower than that (115 kPa) in the study of Mena [30]. This may be due to the following two reasons: 1) due to the possible difference in diagnostic parameters between different brands of machines; 2) because only category 4 nodules were selected for this sample and because category 4 nodules were less likely to have nodules with calcification in this sample, there is a study that suggests that SWE should be used to avoid measurement of calcification in the process of measuring [26]. Zhang [31] also found that both the sensitivity and accuracy of SWE combined with ACR TI-RADS for the diagnosis of thyroid nodules were greatly improved.

In this study, CEUS and SWE quantitative parameters were combined to construct a diagnostic model, and they were subjected to binary logistic regression analysis with ACR TI-RADS scores (*p* < 0.05). The diagnostic efficacy of the diagnostic model (AUC = 0.926) was significantly higher than that of Emax (AUC = 0.816), ΔPE (AUC = 0.819), or ACR TI-RADS score alone (AUC = 0.746). The diagnostic model also had better diagnostic efficacy (AUC = 0.890) than the individual values in the validation group: Emax (AUC = 0.760), ΔPE (AUC = 0.763), and ACR TI-RADS score (AUC = 0.766). Chen et al. [32] found that the diagnostic sensitivity and specificity of contrast-enhanced ultrasound plus SWE were 93.38 and 92.89%, respectively. Wang et al. [19] found that the AUC, sensitivity, specificity, and accuracy of contrast-enhanced ultrasound plus SWE were 0.800, 94, 66, and 83.5%, respectively. Huang [33] found that the AUC, sensitivity, and specificity of contrast-enhanced ultrasound plus SWE were 0.937, 91.67, and 95.65%, respectively. In this study, the diagnostic model had a sensitivity and specificity of 87.50 and 86.80%, respectively. One possible reason for the low sensitivity is that only TI-RADS 4 nodules were included in this study, while high-risk nodules with high malignancy were included in the above studies. Moreover, the accuracy of the model (80%) was higher than that of Emax (67.27%), ∆PE (78.18%), or ACR TI-RADS score (58.18%). The quantitative parameters of contrast-enhanced ultrasound in this study could display the status of nodule vascular perfusion in more detail and more intuitively, avoiding some factors influencing the subjectivity of ultrasound doctors. The diagnostic model constructed based on the combination of CEUS and SWE quantitative parameters and ACR TI-RADS scores can improve the diagnostic efficacy for benign and malignant TI-RADS 4 nodules.

This study had the following limitations: 1) it was performed in only one center; 2) the sample was small; and 3) the pathological types of malignant nodules were all thyroid papillary carcinomas, and other types of malignant thyroid tumors were not covered. In the future, we plan to study different types of thyroid nodules and continue to expand the sample size to explore ways to improve the qualitative diagnostic efficacy for thyroid nodules.

## Conclusion

This new multiparameter diagnostic model performs well at distinguishing benign vs malignant TI-RADS 4 thyroid nodules, providing a valuable reference for clinical diagnosis and treatment.

### Supplementary information

Supplementary information

## Data Availability

The data that support the findings of this study are available on request from the corresponding author.

## Abbreviations

CEUS: contrast-enhanced ultrasound
SWE: shear wave elastography
TIC: time intensity curve
